# Association of baseline tumor-infiltrating lymphocytes and cell-cycle regulation markers on prognosis and mortality in patients with advanced breast cancer according to tumor characteristics and treatment type: an observational study

**DOI:** 10.1007/s10549-026-07991-9

**Published:** 2026-06-04

**Authors:** Sauli Vuoti, Minna Saari, Janne Lahti, Kumar Narasimha, Kai Reinikainen

**Affiliations:** 1 University Of Oulu, Pentti Kaiteran Katu 1, Oulu, 90570 Finland; 2 Chembrain LTD, LSK Business Park, Loppusuora 22, Kauhava, 62200 Finland

**Keywords:** Breast neoplasms, Tumor-infiltrating lymphocytes, Biomarkers, Tumor, Cell proliferation, Ki-67 Antigen, Minichromosome maintenance complex component 2, Disease-free survival, Molecular subtypes

## Abstract

**Background:**

Tumor proliferation and immune infiltration are key determinants of breast cancer biology, yet their prognostic value in the advanced setting remains incompletely defined. We evaluated the clinical relevance of tumor‑infiltrating lymphocytes (TILs) and four cell cycle regulation biomarkers—Ki67, MCM2, Cyclin A, and PHH3—across major breast cancer subtypes in a contemporary real‑world cohort.

**Methods:**

We conducted a retrospective analysis of the outcomes of 398 patients with advanced breast cancer treated between 2020 and 2024. Clinicopathological variables were compared across HER2−/HR+, HER2+, and triple‑negative breast cancer (TNBC). Progression‑free survival (PFS) was assessed using Kaplan–Meier analysis and log‑rank tests. Multivariable polytomous logistic regression identified factors associated with PFS < 2 years. Subtype‑specific associations between TILs and PFS > 2 years were evaluated using multivariable Cox models adjusted for age, ECOG status, tumor grade, and prior therapies. Additional Cox regression models assessed predictors of overall survival (OS).

**Results:**

Low TIL density was independently associated with early progression (RRR 2.28, 95% CI 1.19–4.03). Proliferation markers showed consistent associations with PFS: elevated Ki67, MCM2, Cyclin A, and PHH3 each correlated with shorter survival, with MCM2 showing the strongest effect. TIL–PFS associations were subtype‑dependent. In HR+/HER2 − disease, high TILs were linked to shorter PFS (HR 2.27, 95% CI 1.18–4.02), whereas in TNBC, low TILs predicted markedly worse outcomes (HR 2.33, 95% CI 1.88–2.86). TIL levels were not prognostic in HER2 + disease. Across all subtypes, high expression of Ki67, MCM2, Cyclin A, and PHH3 was significantly associated with reduced OS in multivariable models.

**Conclusions:**

Subtype‑specific immune infiltration and elevated proliferation activity are key predictors of disease trajectory in advanced breast cancer. TILs carry divergent prognostic meaning across subtypes, whereas proliferation markers consistently identify high‑risk disease. Integrating immune and proliferative biomarkers may enhance risk stratification and guide treatment tailoring, particularly within TNBC and hormonally driven tumors.

## Background

Histologically assessed tumor-infiltrating lymphocytes (TILs) have provided significant value in evaluating disease prognosis across several solid tumor types, including lung, ovarian, colorectal, renal cell carcinoma, prostate, and head and neck cancers [1, 2]. The presence of TILs in these tumors has been consistently associated with improved prognosis, suggesting their potential role in predicting treatment response and guiding treatment selection [3, 4]. Despite the historical perception that breast cancer is less immunologically active compared to tumors such as melanoma, recent studies have highlighted the prognostic significance of TILs in breast cancer, particularly in triple-negative breast cancer (TNBC) and HER-2 positive subtypes [5, 6].

In breast cancer, TILs are notably associated with disease-free survival and overall survival, especially in TNBC and HER-2 + subtypes [7, 8]. This association underscores the importance of TILs as a biomarker for prognosis and their potential utility in predicting response to neoadjuvant chemotherapy across all molecular subtypes of breast cancer [9, 10]. The predictive value of TILs in breast cancer has been supported by numerous studies, which have demonstrated the correlation between TIL presence and improved clinical outcomes [1, 2, 11].

Furthermore, cell-cycle regulation markers (CCRs) such as Ki67, Cyclin A, MCM2, and PHH3 have been identified as indicators of poor prognosis when overexpressed [12, 13]. These markers play crucial roles in cell proliferation and tumor progression, making them valuable targets for prognostic evaluation. However, large prospective analyses that integrate both TIL and CCR markers, complemented by long-term follow-up and real-life clinical outcomes, remain scarce [3, 4, 14].

Despite these advancements, there is currently no consensus on clinical guidelines for utilizing these predictive biomarkers in clinical decision-making. The variability in TIL and CCR assessment methods and the lack of standardized protocols contribute to this challenge [5, 6, 15]. Research highlights the need for standardized approaches to biomarker evaluation to enhance their clinical utility [12, 16].

Recent studies have also explored the molecular mechanisms underlying the interaction between TILs and CCRs in the tumor microenvironment. For instance, the role of immune checkpoints and their inhibitors in modulating TIL activity has been a focus of research, with promising results in enhancing anti-tumor immunity [17, 18]. Additionally, the integration of multi-omics approaches, including genomics, transcriptomics, and proteomics, has provided a more comprehensive understanding of the tumor-immune landscape [19, 20]. These advancements have the potential to refine the prognostic and predictive value of TILs and CCRs in breast cancer [13, 14].

In conclusion, while the presence of TILs and the expression of CCRs offer promising prognostic information for patients with advanced breast cancer, further research is needed to establish standardized guidelines for their clinical application [7, 8]. The integration of these biomarkers into routine clinical practice could potentially improve treatment selection and patient outcomes [6, 9]. Continued efforts to standardize biomarker assessment and incorporate novel molecular insights will be crucial in advancing the field of breast cancer prognosis and treatment [10, 15].

In this study, we analyzed data from a cohort of 398 women with advanced breast cancer (ABC) who were undergoing evaluation for routine clinical treatment or participation in clinical trials, with the aim of investigating the prognostic significance of TILs and CCRs in relation to breast cancer recurrence and mortality risk.

## Methods

### Study design and population

This prospective, multicenter cohort study “LEAKED-ABC” was conducted using tumor samples collected from women diagnosed with advanced breast cancer (ABC). Eligible patients were evaluated either for routine clinical treatment or for participation in clinical trials. Inclusion criteria required the availability of histological material obtained from surgical resection or biopsy of regional or distant recurrence, excluding lymphoid tissue metastases. Biomarker analyses (TILs and CCRs) at the time of ABC diagnosis were made with fresh tumor samples.

The patients were followed for up to 5 years, and data from their health records was reviewed retrospectively at the end of the study period. The reviewed data included detailed clinicopathological information such as histological subtype, tumor grade, hormone receptor status, HER2 status, clinical stage at diagnosis, treatment history, recurrence characteristics (time and site), and follow-up data. Patients with ipsilateral in-breast recurrences were excluded due to the difficulty in distinguishing between true recurrence and new primary tumors. ABC was defined as the first diagnosis of either locally recurrent, locally advanced, or metastatic disease.

A total of 442 patients were initially reviewed. Of these, 217 were classified as hormone receptor-positive/HER2-negative (HR+/HER2−), 124 as HER2-positive (HER2+; defined as immunohistochemistry [IHC] score 3 + or IHC 2 + with confirmation by fluorescence in situ hybridization [FISH]), and 101 as triple-negative breast cancer (TNBC). Among the 442 patients, 398 had complete pathology reports and other data available and were included in the final analysis.

Exclusion criteria included incomplete pathology reports, missing clinical endpoint data, prior systemic therapy for metastatic breast cancer, or a diagnosis of another malignancy within five years prior to the ABC diagnosis.

The study was approved by the local ethics committee and classified as an observational study. The primary objective was to evaluate the prognostic significance of TILs and CCRs in relation to progression-free survival (PFS) and response to drug therapy in patients with advanced breast cancer.

### TILs evaluation

Hematoxylin and eosin-stained (HES) slides were retrieved from the biobank. Stromal TILs were assessed according to consensus guidelines [21, 22] by three independent pathologists blinded for clinical data. The average value was used for analyses.

### CCRs evaluation

Immunohistochemical staining for the proliferation markers Ki67, MCM2, Cyclin A, and PHH3 was performed following protocols previously established in the literature [23]. Three independent observers evaluated the expression of these markers. Each slide was examined under 400× magnification, and approximately 500 tumor cells were manually counted to determine the percentage of positively stained nuclei. For Ki67, MCM2, and Cyclin A, nuclear staining of any intensity was considered indicative of positivity, consistent with prior methodologies [24, 25]. Staining intensity was assessed in accordance with the criteria proposed by Nielsen and colleagues [26].

In the case of PHH3, in addition to calculating the proportion of positive cells as described above, mitotic activity was quantified by counting mitotic figures in 10 high-power fields (HPFs) at 400× magnification, as outlined by Bossard et al. [27]. Only nuclei exhibiting strong, condensed chromatin staining—corresponding to mitotic phases such as prophase, metaphase, anaphase, and telophase—were included in the count. Nuclei with faint or diffuse granular staining, indicative of interphase, were excluded from analysis.

### Statistical methods

Spearman’s rank correlation was used to assess associations between non-parametric variables, and Pearson’s chi-squared (χ²) tests were applied for categorical comparisons, including differences in achieving progression-free survival (PFS) of less than 2 years. Kaplan–Meier survival curves were used to estimate overall survival (OS) and progression-free survival (PFS) calculated from the time of the diagnosis of ABC, with differences between groups assessed using the log-rank test.

Multivariable polytomous logistic regression was employed to estimate adjusted relative risk ratios (RRRs) and 95% confidence intervals (CIs) for factors associated with PFS < 2 years. Variable selection combined clinical relevance with backward elimination (*p* < 0.1) to minimize overfitting. Cox proportional hazards models were applied to identify factors associated with OS, and PFS < 2 years. Proportional hazards assumptions were verified using Schoenfeld residuals, and hazard ratios (HRs) with 95% CIs were reported.

All models were adjusted for age, Eastern Cooperative Oncology Group (ECOG) performance status, tumor grade, and prior therapies before advanced breast cancer diagnosis. Analyses were performed using R (version 4.5.2) with the survival and nnet packages. For the markers we used cut-off points described earlier by The International Ki67 in Breast Cancer Working Group (IKWG) recommendations for Ki67 (30%), International TILs Working Group for TILs (30%) and for the other markers we applied earlier published prognostic cut-off points [28] as follows: MCM2 30%, Cyclin A 10%, PHH3 13 mitosis/10 NNL. To evaluate the joint effect of TIL status and proliferation, we fit a multivariable Cox model including TIL (High vs. Low), a categorical count of high proliferation markers (0, 1, ≥ 2), and—where specified—a TIL×count interaction, adjusting for age, ECOG, tumor grade, prior therapies, and subtype.

## Results

### Clinicopathological characteristics

Between 2020 and 2024, a total of 398 patients with advanced breast cancer were included in the analysis. Among these, 206 (51.8%) were classified as HER2−/HR+, 102 (25.6%) as HER2+, and 90 (22.6%) as triple-negative breast cancer (TNBC). Baseline characteristics are summarized in Table 1. The median age at diagnosis was 64.7 years (range 39–83) for HER2−/HR+, 63.2 years (range 38–79) for HER2+, and 61.6 years (range 37–75) for TNBC. The majority of patients across all subgroups were Caucasian (> 94%).

Performance status was generally favourable, with ECOG 0 observed in 58.5% of HER2−/HR+, 59.6% of HER2+, and 54.6% of TNBC patients. Disease setting at advanced stage was similar among groups: de novo metastatic disease accounted for 41.0% in HER2−/HR+, 44.0% in HER2+, and 39.8% in TNBC, while metastatic recurrence was slightly less frequent in HER2+ (51.9%) compared to HER2−/HR+ (54.5%) and TNBC (54.1%). Locoregional recurrence was uncommon (< 6% in all groups).

Visceral metastases were the most common site of spread, affecting 48.6% of HER2−/HR+, 49.3% of HER2+, and 50.1% of TNBC patients. Bone-only metastases were less frequent in HER2−/HR+ (21.3%) compared to HER2+ (22.9%) and TNBC (23.4%). Other metastatic sites accounted for approximately 26–30% across subgroups.

Prior systemic therapy patterns differed substantially. Neoadjuvant or adjuvant chemotherapy was reported in 40.6% of HER2−/HR+, 75.3% of HER2+, and 85.5% of TNBC patients. Endocrine therapy before advanced disease was common in HER2−/HR+ (84.7%) but less frequent in HER2+ (57.1%) and rare in TNBC (5.7%).

Histological grade distribution showed that high-grade tumors (Grade III) predominated in TNBC (72.8%) and HER2+ (70.0%), whereas HER2−/HR + had a more balanced profile (Grade I: 15.4%, Grade II: 51.0%, Grade III: 33.6%).

**Table 1:** Patient characteristics stratified by biological subgroup and cancer stage

CHARACTERISTICS	HER2-/HR+	HER2+	TNBC
**No. of patients**	206	102	90
**Median age**,** years (range)**	64.7 (39–83)	63.2 (38–79)	61.6 (37–75)
**Race (%)**			
Caucasian	96.1	96.8	94.5
Other	3.9	3.2	5.5
**ECOG performance status, No. (%)**			
0	58.5	59.6	54.6
1	39.5	39.4	42.2
2	2.0	1	3.2
**Disease setting, No. (%)**			
De novo metastatic	41.0	44.0	39.8
Metastatic recurrent	54.5	51.9	54.1
Locoregionally recurrent	4.5	4.1	6.1
**Metastatic site, No. (%)**			
Visceral	48.6	49.3	50.1
Bone only	21.3	22.9	23.4
Other	30.1	27.8	26.5
**Prior neoadjuvant or adjuvant chemotherapy, No. (%)**			
Yes	40.6	75.3	85.5
No	59.4	24.7	14.5
**Prior endocrine therapy, No.**			
Yes	84.7	57.1	5.7
No	15.3	42.9	94.3
**Grade**			
I	15.4	3.8	8.8
II	51.0	26.2	18.4
III	33.6	70.0	72.8
**LN status**			
Negative	18.7	6.4	4.3
Positive	81.3	93.6	95.7

### Multilevel risk factors for PFS < 2 years

**Fig. 1:**
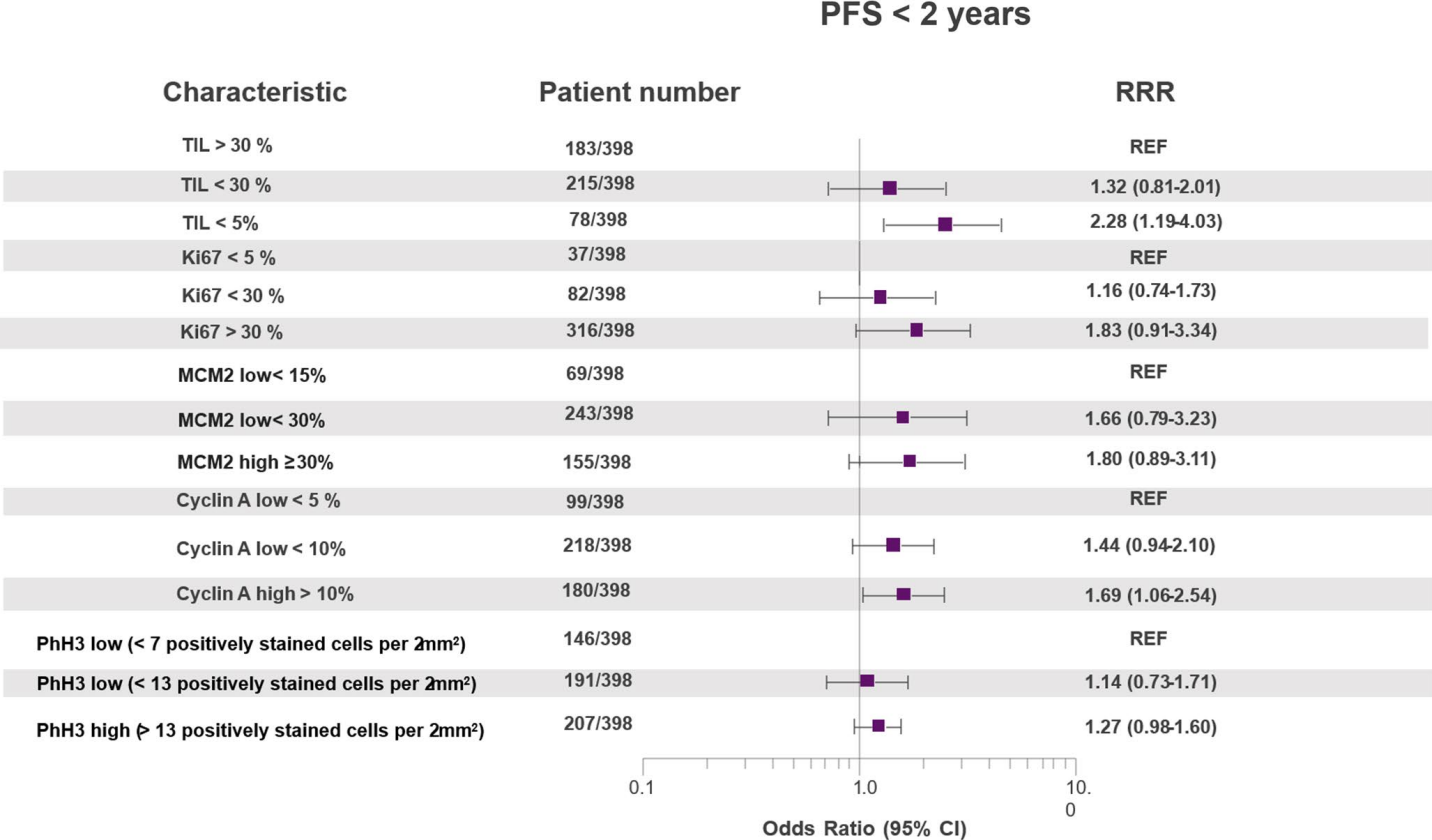
Multilevel risk factors for PFS < 2 years among the full breast cancer patient population. For individual-level and clinical factors, multivariable polytomous logistic regressions were adjusted for age, ECOG, tumor grade and previous therapies before the advanced breast cancer stage

Multivariable polytomous logistic regression was performed to identify individual-level and clinical factors associated with progression-free survival (PFS) of less than 2 years among the full breast cancer cohort (*n* = 398), adjusting for age, ECOG performance status, tumor grade, and prior therapies before advanced disease. A threshold of 2 years was selected to define early progression because it represents a clinically meaningful benchmark for durable disease control in advanced breast cancer and aligns with prior literature. This cut-off also mitigates bias related to limited follow-up duration in our cohort. The analysis revealed that high tumor-infiltrating lymphocytes (TIL > 30%) served as the reference category, while patients with TIL < 5% had a significantly increased risk of early progression (RRR = 2.28, 95% CI: 1.19–4.03). Similarly, Ki67 > 30% was associated with higher odds of PFS < 2 years compared to Ki67 ≤ 30% (RRR = 1.83, 95% CI: 0.91–3.34), although this did not reach statistical significance. For replication licensing markers, MCM2 ≥ 30% showed an elevated risk (RRR = 1.80, 95% CI: 0.89–3.11) relative to MCM2 < 30%. Cyclin A > 10% was also linked to increased risk (RRR = 1.69, 95% CI: 1.06–2.54). In contrast, PHH3 positivity demonstrated only modest associations, with PHH3 > 13 cells per 2 mm² yielding an RRR of 1.27 (95% CI: 0.98–1.60) compared to the lowest category. Overall, proliferation-related biomarkers (Ki67, MCM2, Cyclin A) and low TIL density emerged as key predictors of early progression, whereas PHH3 showed weaker associations (Fig. 1).

**Fig. 2:**
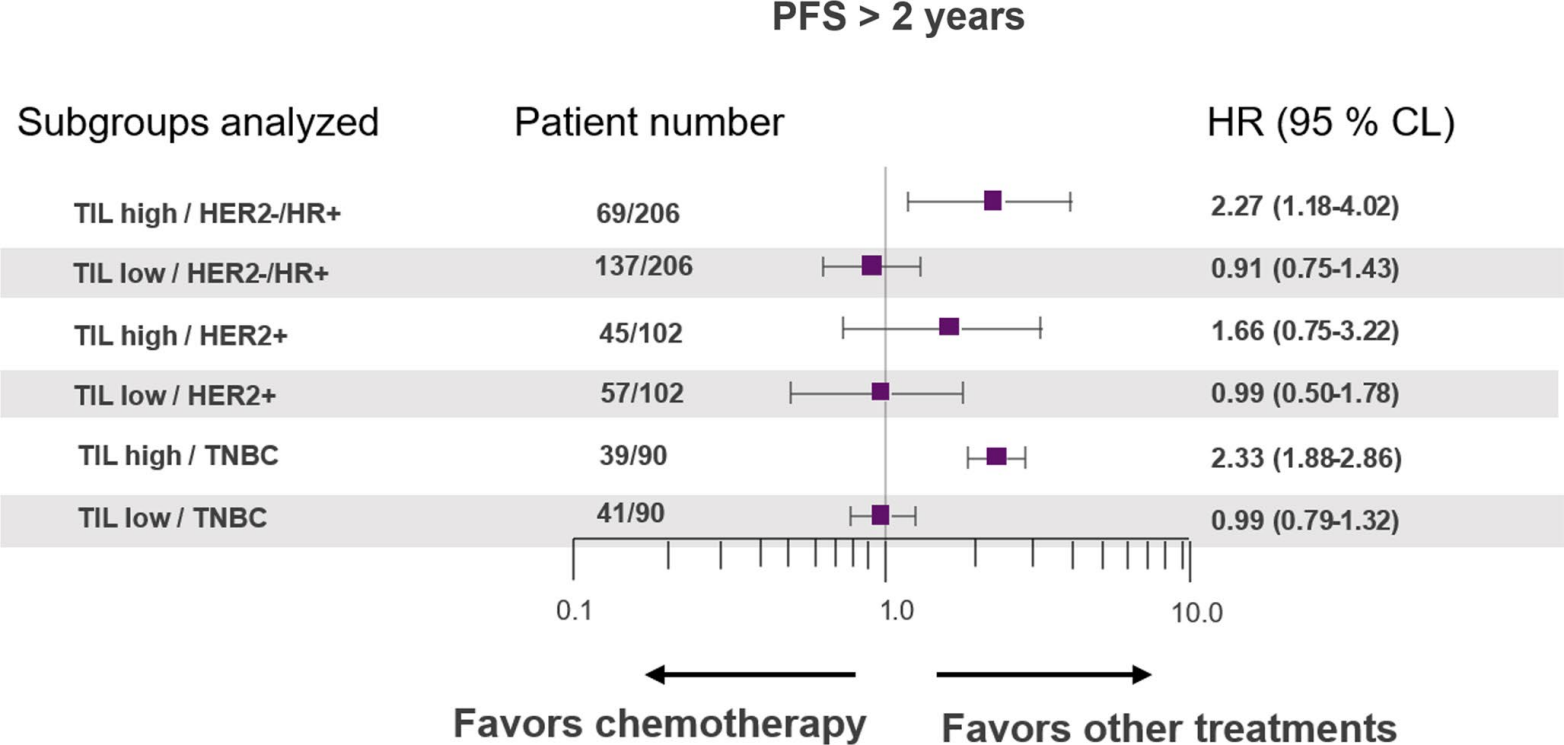
Multivariable Cox regression model of factors associated with PFS of over 2 years. Hazard ratios (HRs) are estimated from Cox models adjusted for age, ECOG, tumor grade, and prior therapies. HRs compare chemotherapy‑first vs. other initial treatments within each TIL–subtype subgroup; HR < 1 indicates lower hazard (favors chemotherapy), HR > 1 indicates higher hazard (favors other treatments). Treatment‑by‑subgroup interaction p‑values are provided to assess heterogeneity of treatment effect. The other treatments group included: ADC conjugates, immunotherapies, CDK 4/6 inhibitors, monoclonal antibodies, mTOR inhibitors and PARP inhibitors as initial therapy for ABC

Using multivariable Cox models adjusted for age, ECOG performance status, tumor grade, and prior therapies, we evaluated the association of tumor-infiltrating lymphocytes (TILs) with achieving PFS > 2 years across molecular subgroups (Fig. 2). In HER2−/HR+ disease, high TILs (> 30%, *n* = 69/206) were associated with a higher hazard of progression compared with low TILs (< 30%, HR = 2.27, 95% CI 1.18–4.02, *p* = 0.0088). In contrast, low TILs (*n* = 137/398) within HER2−/HR+ showed no significant association (HR = 0.91, 95% CI 0.75–1.43, *p* = 0.567). For HER2 + disease, neither low TILs (*n* = 45/102; HR = 1.66, 95% CI 0.75–3.22, *p* = 0.173) nor high TILs (*n* = 57/102; HR = 0.99, 95% CI 0.50–1.78, *p* = 0.975) were significantly associated with the outcome. In TNBC, low TILs (*n* = 39/90) were associated with a higher hazard (HR = 2.33, 95% CI 1.88–2.86, *p* = 2.7 × 10⁻¹⁵), whereas high TILs (*n* = 41/90) were not significantly associated (HR = 0.99, 95% CI 0.79–1.32, *p* = 0.939).

**Fig. 3:**
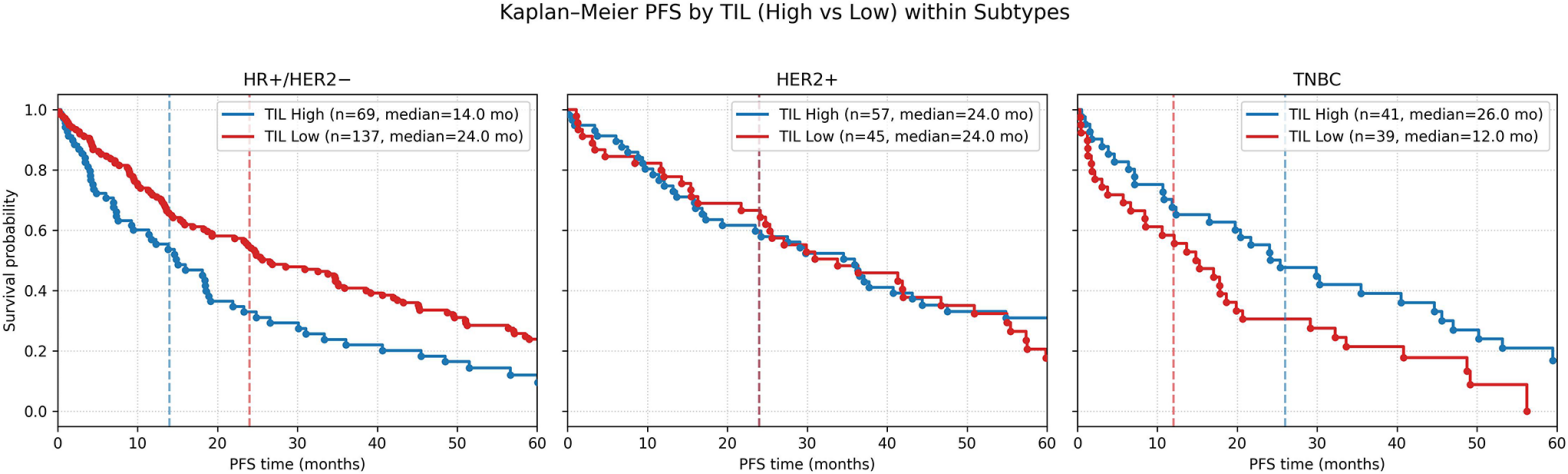
Kaplan-Meier analysis of PFS according to TIL categories and breast cancer subtypes. HR+/HER2; Log‑rank: χ² = 13.29, *p* = 0.00027, Cox HR (High vs. Low): 1.82 (95% CI 1.31–2.52), *p* = 0.00033. HER2+; Log‑rank: χ² = 0.46, *p* = 0.499, Cox HR (High vs. Low): 0.86 (95% CI 0.56–1.33), *p* = 0.499, TNBC; Log‑rank: χ² = 4.81, *p* = 0.0283, Cox HR (High vs. Low): 0.574 (95% CI 0.347–0.948), *p* = 0.0302

Kaplan–Meier analysis demonstrated distinct PFS patterns for TIL high versus TIL low groups across breast cancer subtypes (Fig. 3). In HR+/HER2 − disease, patients with high TILs had a median PFS of 14.0 months, compared to 24.0 months for those with low TILs. The log-rank test indicated a statistically significant difference (χ² ≈ 13.3, *P* < 0.001), and Cox regression confirmed an increased hazard for TIL-high patients (HR ≈ 1.82, 95% CI 1.31–2.52, *P* < 0.001). In HER2 + disease, median PFS was similar between TIL-high and TIL-low groups (24.0 months for both), with no significant difference by log-rank (χ² ≈ 0.46, *P* = 0.50) or Cox analysis (HR ≈ 0.86, 95% CI 0.56–1.33, *P* = 0.50). In TNBC, patients with high TILs achieved a median PFS of 26.0 months, compared to 12.0 months for those with low TILs. This difference was statistically significant (log-rank χ² ≈ 4.81, *P* = 0.028), and Cox regression showed a reduced hazard for TIL-high patients (HR ≈ 0.57, 95% CI 0.35–0.95, *P* = 0.030).

### Progression-free survival analysis by biomarker

**Fig. 4:**
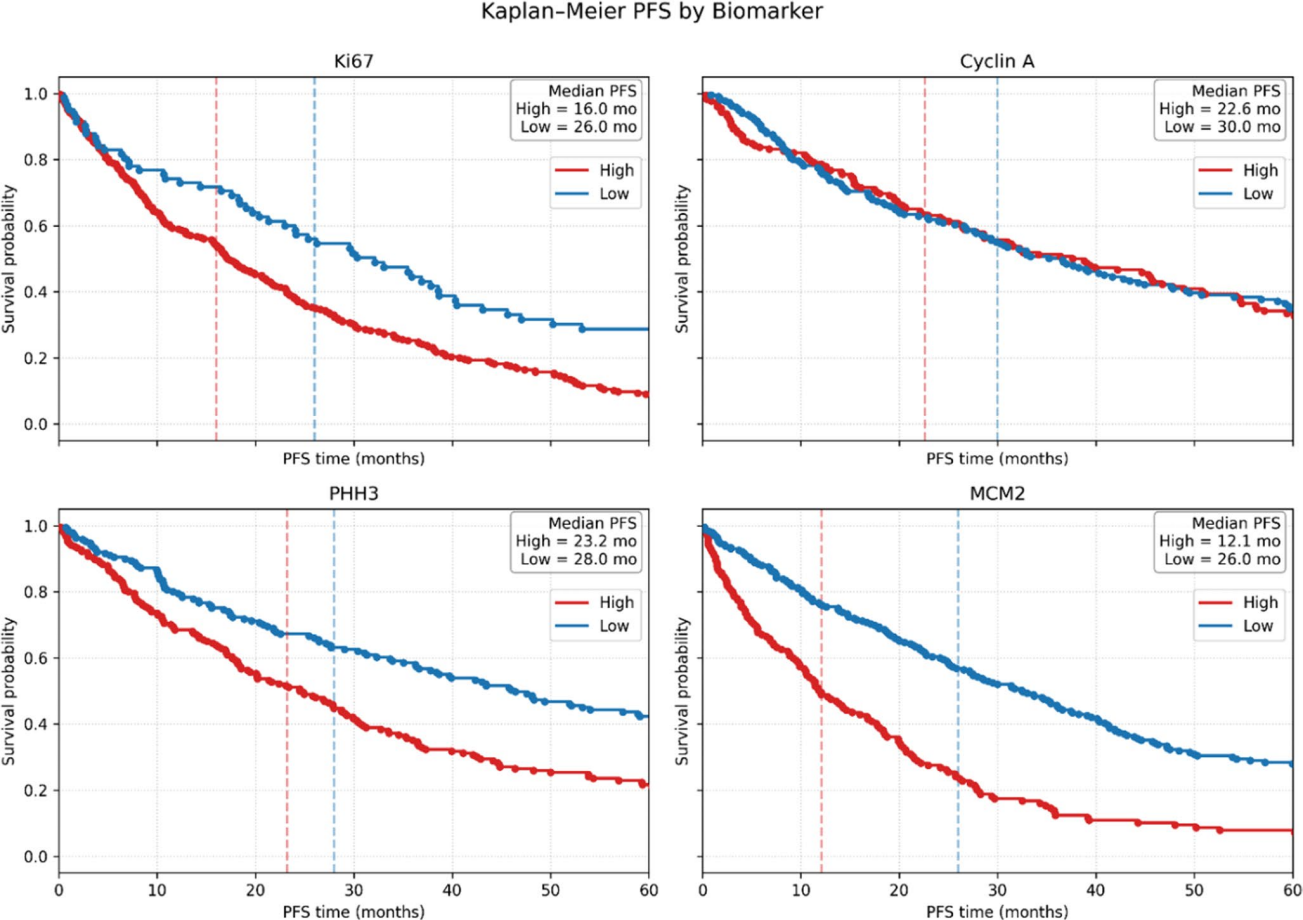
Kaplan-Meier analysis of PFS according to biomarker

For Ki67, the median PFS was 16.0 months for patients with high Ki67 and 26.0 months for those with low Ki67 (log-rank χ² = 16.28, *P* = 0.0001). The overall survival curves demonstrated a clear separation favoring the low Ki67 group (Fig. 4). For Cyclin A, the median PFS was 22.6 months in the high-expression group and 30.0 months in the low-expression group (log-rank χ² = 3.32, *P* = 0.0685), indicating a non-significant trend toward improved PFS in patients with lower Cyclin A levels. For PHH3, patients with high PHH3 had a median PFS of 23.2 months, compared to 28.0 months in the low PHH3 group (log-rank χ² = 5.56, *P* = 0.0184), suggesting a statistically significant association between lower PHH3 expression and longer PFS. For MCM2, the difference was most pronounced: median PFS was 12.1 months for high MCM2 versus 26.0 months for low MCM2 (log-rank χ² = 35.43, *P* < 0.0001), confirming MCM2 as a strong predictor of early progression.

### Factors associated with overall survival (Multivariable Cox model)

In the multivariable Cox regression model for overall survival (OS), Table 2, age at index was independently associated with shorter survival (HR 1.09, 95% CI 1.08–1.09, *p* < 0.001). Using HR+/HER2 − as the reference subgroup, both HER2+ (HR 1.44, 95% CI 1.13–1.63, *p* < 0.001) and TNBC (HR 1.89, 95% CI 1.68–2.15, *p* < 0.001) were associated with increased hazard of death. Positive lymph node status at baseline conferred worse OS compared with node-negative disease (HR 1.26, 95% CI 1.11–1.49, *p* = 0.001). For immune infiltration, low TIL levels were associated with inferior OS relative to high TILs (HR 1.30, 95% CI 1.12–1.50, *p* = 0.001). Across proliferation markers, higher expression was consistently associated with poorer OS: Ki67 high (HR 1.75, 95% CI 1.37–2.24, *p* < 0.001), MCM2 high (HR 1.85, 95% CI 1.50–2.28, *p* < 0.001), Cyclin A high (HR 1.24, 95% CI 1.12–1.50, *p* < 0.001), and PHH3 high (HR 1.29, 95% CI 1.15–1.55, *p* = 0.001).

**Table 2:** Factors associated with overall survival: Multivariable Cox regression analysis

Variable	HR (95% CL)	*p* value
**Age at index**	1.09 (1.08–1.09)	< 0.001
**Subgroup**		
HER2-/HR+	1.00	
HER2+	1.44 (1.13–1.63)	< 0.001
TNBC	1.89 (1.68–2.15)	< 0.001
**LN Status**		
Negative	1.00	
Positive	1.26 (1.11–1.49)	0.001
**TIL levels**		
High	1.00	
Low	1.30 (1.12–1.50)	0.001
**Ki67**		
Low	1.00	
High	1.75 (1.37–2.24)	< 0.001
**MCM2**		
Low	1.00	
High	1.85 (1.50–2.28)	< 0.001
**Cyclin A**		
Low	1.00	
High	1.24 (1.12–1.50)	< 0.001
**PHH3**		
Low	1.00	
High	1.29 (1.15–1.55)	0.001
TIL High (> 30%) + 1 of the other biomarkers high*	1.45	< 0.001
TIL High (> 30%) + ≥2 of the other biomarkers high*	2.05	< 0.001
TIL Low (< 30%) + 1 of the other biomarkers high*	1.90	< 0.001
TIL Low (< 30%) + ≥2 of the other biomarkers high*	2.70	< 0.001

*Composite proliferation burden was defined as the count of high markers among PHH3, MCM2, Ki67, and Cyclin A (cutoffs as specified in Methods). Rows labeled ‘TIL High/Low + 1 high’ and ‘TIL High/Low + ≥ 2 high’ reflect joint categories combining TIL status with the number of high proliferation markers. Hazard ratios are adjusted for age, ECOG, tumor grade, prior therapies before the advanced stage, and molecular subtype. The reference for these rows is TIL High + 0 high proliferation markers

## Discussion

In this study of 398 patients with advanced breast cancer, we identified distinct clinicopathological and biomarker-related factors associated with progression-free survival (PFS) and overall survival (OS). Our findings reinforce the prognostic heterogeneity across molecular subtypes and highlight the potential role of proliferation markers and immune infiltration in shaping outcomes.

Consistent with prior reports, TNBC and HER2 + subtypes were associated with significantly worse OS compared to HR+/HER2 − disease, reflecting their aggressive biology and limited endocrine responsiveness [29, 30]. Positive lymph node status and increasing age also emerged as independent adverse prognostic factors, in line with established clinical predictors [31].

Immune infiltration, assessed by tumor-infiltrating lymphocytes (TILs), demonstrated subtype-dependent associations. In TNBC, low TIL density was strongly linked to early progression and inferior survival, supporting previous evidence that high TILs predict better chemotherapy response and improved outcomes in this subtype [29, 32]. Conversely, in HR+/HER2 − disease, high TILs were associated with shorter PFS, a finding reported in other studies suggesting that immune activation in luminal tumors may reflect aggressive biology rather than therapeutic benefit [33]. For HER2 + disease, TILs showed no significant prognostic impact in our cohort, which contrasts with early-stage studies where high TILs predict benefit from anti-HER2 therapy [34]. This discrepancy may relate to advanced disease setting, treatment heterogeneity, and smaller subgroup sizes.

Proliferation-related biomarkers (Ki67, MCM2, Cyclin A, PHH3) consistently correlated with poor outcomes, both for PFS and OS. Among these, MCM2 demonstrated the strongest association, aligning with its role as a replication licensing factor and a marker of aggressive tumor biology [35]. Ki67 also showed robust prognostic value, confirming its utility in advanced disease despite ongoing debate about optimal cut-offs [36]. Cyclin A and PHH3 exhibited weaker but significant associations, suggesting that mitotic activity contributes to progression risk but may be less discriminatory than licensing markers. Regardless of TIL levels, high levels of more than one proliferation-related biomarker increased the risk notably.

Our multivariable models adjusted for key clinical covariates, and proportional hazards assumptions were verified. The observed patterns underscore the interplay between tumor biology and immune contexture in advanced breast cancer. While proliferation markers primarily reflect intrinsic aggressiveness, TILs may indicate differential sensitivity to systemic therapies, including chemotherapy and emerging immunotherapies. The lack of clear benefit for high TILs in HER2 + disease warrants further investigation, particularly in relation to anti-HER2 regimens and immune checkpoint inhibitors.

This work has several limitations. These include potential treatment heterogeneity, partly retrospective design (review of patient health records retrospectively), and natural variety among scoring TIL and biomarker results. Additionally, even if the treating oncologists were not aware of the biomarker or TIL levels at the time of selecting the treatment, clinical features have most likely influenced treatment decisions and thus created biases in the treatment outcomes. For this reason, the trend towards an improved response to immunotherapies among those TIL high may seem more pronounced than in reality. Follow-up was limited to approximately five years, which may influence long-term survival estimates. Strengths of this study include a well-characterized cohort and integration of multiple biomarkers in our analysis with real-world outcomes and treatments carried out in a real-world setting.

Our findings support incorporating proliferation markers and immune profiling into risk stratification for advanced breast cancer. MCM2 and Ki67 may help identify patients at high risk for early progression, while TIL assessment could inform therapeutic decisions, particularly in TNBC where immunotherapy is an emerging standard. Prospective studies are needed to validate these associations and explore predictive interactions with targeted and immune-based therapies.

## Conclusions

In this cohort of patients with advanced breast cancer, we identified both clinical and biological factors that independently influence progression-free and overall survival. Our findings highlight the substantial prognostic heterogeneity across breast cancer subtypes and underscore the relevance of tumor proliferation activity and immune microenvironment in shaping patient outcomes.

Collectively, these findings suggest that integrating TIL assessment with granular CCR profiling may improve risk stratification in advanced breast cancer. While CCRs primarily reflect intrinsic tumor aggressiveness, immune infiltration may capture aspects of treatment responsiveness that are unique to each subtype.

Further prospective studies are warranted to validate these observations and evaluate the potential clinical utility of combining immune and proliferation biomarkers to guide therapeutic decisions, including the use of immunotherapies, targeted agents, and treatment intensification strategies tailored to biological risk.

## Data Availability

Deidentified data used in this study are available from the corresponding author upon reasonable request.
